# Uncovering the Differences: How DLQI and WHO-5 Scores Vary in Moderate-to-Severe Psoriasis Patients Treated with Tildrakizumab 100 mg vs. 200 mg?

**DOI:** 10.3390/jcm13175240

**Published:** 2024-09-04

**Authors:** Emanuele Trovato, Martina Dragotto, Eugenio Capalbo, Alessandra Cartocci, Pietro Rubegni, Laura Calabrese

**Affiliations:** 1 Dermatology Unit, Department of Medical, Surgical and Neurological Sciences, University of Siena, 53100 Siena, Italy; trovato.ema@gmail.com (E.T.);; 2 Department of Medical Biotechnology, University of Siena, 53100 Siena, Italy; 3 Dermatologia, Dipartimento di Medicina e Chirurgia Traslazionale, Università Cattolica del Sacro Cuore, 00168 Rome, Italy

**Keywords:** biologics, psoriasis, quality of life, tildrakizumab, patient-reported outcomes, DLQI, WHO-5

## Abstract

**Background/Objectives:** Psoriasis (PsO) is a chronic inflammatory skin disease that severely impacts patients’ quality of life (QoL). Its global prevalence is about 2%, with significant regional variations. PsO manifests in the form of erythematous and scaly plaques, causing intense pruritus and discomfort and limiting daily activities. The condition often includes comorbidities such as psoriatic arthritis, cardiovascular diseases, and metabolic syndrome, further deteriorating QoL. Psychological well-being is notably affected, with high levels of depression and anxiety due to the visible lesions, leading to social stigma and isolation. QoL indexes like WHO-QoL and SF-36 assess various well-being aspects, while patient-reported outcomes (PROs) provide a comprehensive understanding of PsO’s impact. However, there are no universally shared PROs in outpatient practice to fully understand the impact of the disease and associated therapies. This study aims to evaluate differences between DLQI and WHO-5 in adult patients with moderate-to-severe PsO treated with tildrakizumab 100 mg or 200 mg. **Methods:** The study was conducted at the University Hospital of Siena, Italy, from May 2023 to April 2024. Data from 15 patients treated with tildrakizumab 200 mg and 15 patients treated with tildrakizumab 100 mg, observed for at least 28 weeks, were recorded. Demographic data, PASI, DLQI, and WHO-5 scores were analyzed. Patients in the 100 mg group (G100) were selected to match the demographic characteristics of the 200 mg group (G200). Reduction rates of DLQI and WHO-5 were assessed at baseline values and after 4, 16, and 28 weeks. **Results**: Both groups experienced improvements in QoL. The group treated with 200 mg showed more pronounced and rapid reductions in DLQI and WHO-5 scores compared to the 100 mg group. WHO-5 demonstrated faster improvements in overall well-being than DLQI, indicating its greater sensitivity to changes in mental well-being and overall QoL. No differences in adverse events were observed between the two groups, with no major adverse events reported. **Conclusions:** In our study, WHO-5 proved more sensitive than DLQI in capturing well-being changes in PsO patients treated with tildrakizumab. However, a combined use of both WHO-5 and DLQI questionnaires should be encouraged in clinical practice. Furthermore, this study confirmed the superior QoL improvement associated with tildrakizumab 200 mg compared to 100 mg. Future research should explore the long-term impact on QoL and comparative effectiveness among other biologic therapies in diverse patient populations.

## 1. Introduction

Psoriasis (PsO) is a chronic inflammatory skin disease that can severely impact patients’ quality of life (QoL). Its prevalence varies across different regions and populations; globally, its prevalence is estimated to be around 2%, with some regions experiencing higher rates [1]. The variability in prevalence suggests that both genetic and environmental factors play a significant role in the pathogenesis of PsO [2]. This chronic condition, which is traditionally recognized for its impact on the skin, has far-reaching effects that extend well beyond dermatological symptoms. In addition to affecting the skin, it can manifest as a serious joint disease known as psoriatic arthritis, which can occur even in the absence of any visible skin lesions. This means that individuals may suffer from joint pain, stiffness, and swelling without the telltale signs of psoriasis on their skin, making diagnosis and treatment more challenging. The dual nature of this condition—as both a skin and joint disease—amplifies its complexity, contributing to significant physical discomfort and disability. Moreover, the emotional and psychosocial burdens associated with managing a chronic, unpredictable, and often misunderstood illness are profound, further impacting the quality of life of those with such conditions. These multifaceted challenges underscore the critical need for continued research into the underlying mechanisms of both psoriasis and psoriatic arthritis, as well as the development of more effective, comprehensive healthcare strategies tailored to addressing the diverse manifestations of the disease [3]. The chronic and relapsing nature of the disease is often compounded by comorbid conditions such as psoriatic arthritis, cardiovascular diseases, and metabolic syndrome, which further deteriorate overall health [4]. In terms of physical health, a QoL index assesses aspects like physical functioning, energy levels, pain, and general health status [5]. Psychological well-being is a crucial component, which includes mental health indicators such as depression, anxiety, stress, and emotional balance [6]. Psychologically, PsO is associated with substantial morbidity, including elevated levels of depression, anxiety, and suicidal ideation. The visibility of psoriatic lesions contributes to psychological distress and social stigmatization, exacerbating mental health issues [7]. To assess the impact of the disease on patients’ lives, patient-reported outcomes (PROs) are commonly used and refer to data that come directly from the patient, without interpretation by a clinician or anyone else. These outcomes typically include assessments of symptoms, functional status, health-related quality of life (HRQoL), and satisfaction with treatment [8]. PROs are crucial in providing a comprehensive understanding of the impact of a disease and its treatment on patients’ lives, beyond clinical measures alone [9]. Key PRO instruments commonly used include the Dermatology Life Quality Index (DLQI), which evaluates the disease’s impact on daily activities, relationships, and emotional well-being [10], and the Psoriasis Symptom Inventory (PSI) and the Psoriasis Disability Index (PDI), which further detail symptom severity and functional limitations, respectively [11]. Furthermore, the World Health Organization-Five Well-Being Index (WHO-5) is a widely used self-reported questionnaire designed to measure subjective well-being and psychological health [12]. Improvements in WHO-5 scores following treatment interventions indicate positive changes in patients’ well-being and QoL. To date, there are no universally shared PROs in outpatient practice that allow us to fully understand the impact of the disease and therapy on the patient’s life [13]. Tildrakizumab (Ilumetri^®^) is a humanized monoclonal antibody that specifically targets interleukin-23 (IL-23), a cytokine involved in the inflammatory pathways that contribute to PsO. By inhibiting the p19 subunit of IL-23, tildrakizumab interferes with the IL-23/Th17 axis, which plays a crucial role in the pathogenesis of PsO. Clinically, tildrakizumab is approved for the treatment of moderate-to-severe plaque PsO [14]. Its efficacy has been demonstrated through several phase III clinical trials, which showed significant improvements in disease severity, measured using the the Psoriasis Area and Severity Index (PASI), compared to placebo. Patients receiving tildrakizumab typically exhibit substantial reductions in skin lesions and improved QoL [15,16]. Tildrakizumab (Ilumetri^®^) 200 mg was recently introduced into the therapeutic armamentarium for managing adult patients with moderate-to-severe plaque PsO. According to the technical data sheet, this dosage is currently reserved for patients weighing over 90 kg and with a PASI > 20. Its efficacy has also been confirmed in real-life studies [16,17]. However, there are a paucity of data comparing 100 mg and 200 mg in terms of their impact on patients’ quality of life. The aim of this study was to evaluate the trend of change in the DLQI and WHO-5 in two groups of adult patients with moderate-to-severe PsO treated with tildrakizumab (Ilumetri^®^) 100 mg and 200 mg, to highlight the strengths and/or weaknesses of the two questionnaires and how dual dosing of tildrakizumab may play a role in this regard.

## 2. Materials and Methods

**Figure 1:**
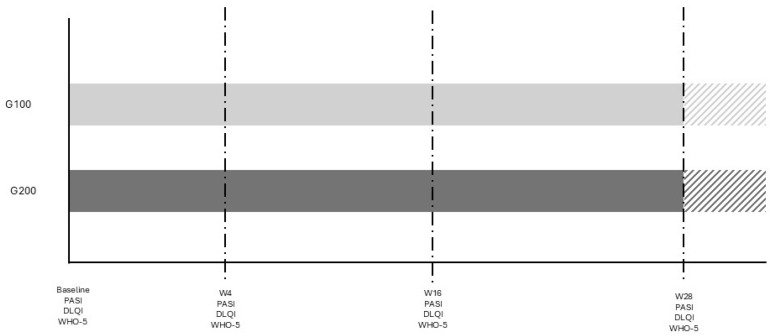
Study diagram. The different color area is referred to patients who continue therapy without any evaluation in the study.

The study was conducted at University Hospital in Siena, Italy, from May 2023 to April 2024. It involved a comparison between two groups of patients with plaque psoriasis: 15 consecutive patients treated with 200 mg tildrakizumab (Ilumetri^®^) and 15 patients treated with 100 mg tildrakizumab (Ilumetri^®^). All patients were observed for a period of at least 28 weeks. The inclusion criteria for the study required patients to be over 18 years of age, have a confirmed diagnosis of plaque psoriasis, and to be eligible for treatment with tildrakizumab 200 mg. Patients with other forms of psoriasis, such as pustular or erythrodermic psoriasis, those who did not complete the 28-week follow-up, or those who withdrew their informed consent were excluded from the study. The tildrakizumab therapy was administered at baseline (time 0), after 4 weeks, and then every 12 weeks, as specified in the drug’s technical data sheet. Demographic data, as well as PASI, DLQI, and WHO-5 scores, were assessed for all patients. The 100 mg group (G100) was selected to match the demographic characteristics of the 200 mg group (G200) in order to obtain comparable data and minimize potential confounding factors. Given the sample size and the study’s objective, the reduction rates of DLQI and WHO-5 were evaluated at baseline and after 4, 16, and 28 weeks in both patient groups. The study adhered to the criteria outlined in the 1964 Declaration of Helsinki and its subsequent amendments, and all participants provided written informed consent for their participation in the study. A study flow diagram is reported in Figure 1.

## 3. Results

**Figure 2:**
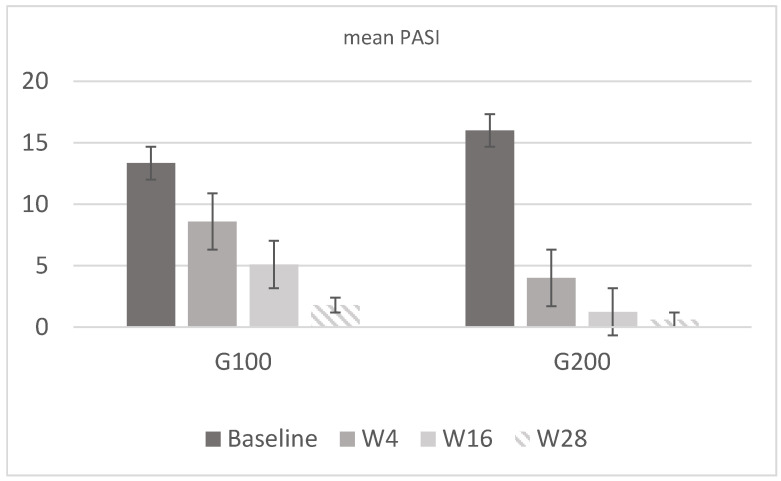
Mean PASI values in G100 and G200. In the first group, the mean value was 13.34 at baseline, 8.6 at w4, 5.1 at w16, and 1.8 at w28. In G200, the value was 16 at baseline, 4 at w4, 1.25 at w16, and 0.6 at w28. Both groups show a marked reduction in PASI scores from baseline to week 28, with the G200 group demonstrating a more rapid and substantial improvement compared to the G100 group.

**Figure 3:**
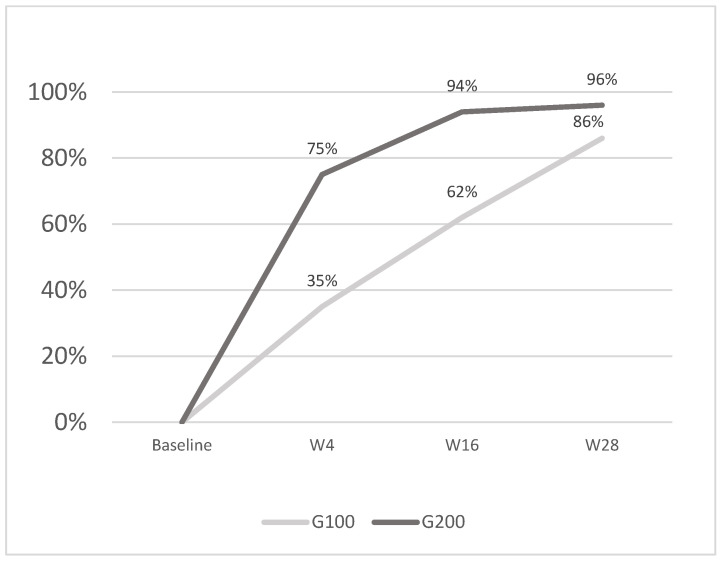
The image shows the average percentage reduction in PASI scores: G100 achieved 35% at w4, 62% at w16, and 86% at w28, while G200 reached 75% at w4, 94% at w16, and 96% at w28. The G200 group demonstrated a faster and greater reduction at each time point compared to G100.

In the 100 mg group (G100), there were eight men (53%) and seven women (47%) with a mean age of 55.2 ± 11 years and a mean weight of 77.6 ± 11.6 kg. In the 200 mg group (G200), there were nine men (60%) and six women (40%), with a mean age of 56 ± 10.8 years and a mean weight of 86.2 ± 10.8 kg. In G100, the PASI score was 13.34 ± 2.35 at baseline, while in G200, it was 16 ± 1.65. Regarding DLQI, in G100, the mean score was 20 ± 1.8 at baseline, 12.8 ± 2.1 at week 4, 5.27 ± 0.6 at week 16, and 2.73 ± 0.5 at week 28; in G200, it was 20.1 ± 1.9 at baseline, 12.27 ± 2.3 at week 4, 2.4 ± 0.7 at week 16, and 1.5 ± 1.1 at week 28. Analyzing the mean WHO-5 score, in G100, it was 17.5 ± 2.9 at baseline, 42.1 ± 4.2 at week 4, 55.2 ± 3.3 at week 16, and 73.9 ± 6.3 at week 28; in G200, it was 18 ± 3.2 at baseline, 60.5 ± 3.7 at week 4, 72.8 ± 6 at week 16, and 88 ± 5 at week 28. The results confirmed what had already been reported in real-life studies, demonstrating a higher efficacy of tildrakizumab 200 mg compared to 100 mg, as reported in Figure 2 and Figure 3. This result is derived only from the mean change in PASI and is not, however, the primary endpoint of the study.

**Figure 4:**
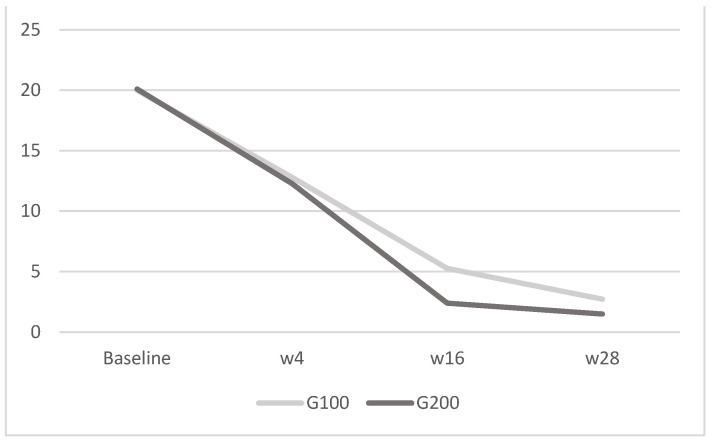
DLQI: in G100, the mean score was 20 ± 1.8 at baseline, 12.8 ± 2.1 at week 4, 5.27 ± 0.6 at week 16, and 2.73 ± 0.5 at week 28; in G200, it was 20.1 ± 1.9 at baseline, 12.27 ± 2.3 at week 4, 2.4 ± 0.7 at week 16, and 1.5 ± 1.1 at week 28. The image depicts the mean DLQI scores for the G100 and G200 groups over time. Both groups experience a decrease in scores from baseline to week 28, with the G200 group showing a more pronounced reduction compared to the G100 group.

**Figure 5:**
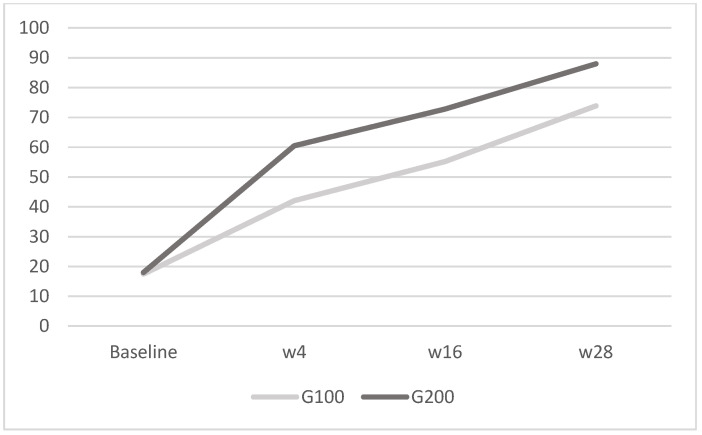
WHO-5: in G100, the mean was 17.5 ± 2.9 at baseline, 42.1 ± 4.2 at week 4, 55.2 ± 3.3 at week 16, and 73.9 ± 6.3 at week 28; in G200, it was 18 ± 3.2 at baseline, 60.5 ± 3.7 at week 4, 72.8 ± 6 at week 16, and 88 ± 5 at week 28. The image shows the mean WHO-5 scores for the G100 and G200 groups over time. Both groups exhibit improvements in scores from baseline to week 28, with the G200 group displaying a more substantial increase compared to the G100 group.

Importantly, the two groups did not differ in terms of recorded adverse effects, and none of the patients reported major adverse events. In relation to QoL assessment, both the DLQI and the WHO-5 values showed a greater reduction in the group receiving 200 mg of tildrakizumab compared to the group receiving 100 mg. Additionally, the WHO-5 exhibited a more rapid reduction in values at 4, 16, and 28 weeks compared to the DLQI. Both groups showed a reduction in DLQI scores over time, indicating an improvement in quality of life; the G200 group had a slightly higher baseline score but demonstrated a greater percentage reduction by week 28, suggesting a more pronounced improvement with the higher dosage. For WHO-5, both groups exhibited substantial increases in WHO-5 scores, reflecting enhanced well-being; the G200 group had consistently higher scores at all time points post-baseline and achieved a higher percentage increase by week 28, indicating a more substantial improvement in mental well-being when taking the higher dosage. Therefore, evaluating the two questionaries, as reported in Figure 4 and Figure 5, the WHO-5 was found to be more prone than the DLQI to reflecting improvements in quality of life. This suggests that WHO-5 may be a more sensitive and responsive tool for capturing changes in well-being over time in patients undergoing treatment with tildrakizumab.

## 4. Discussion

Due to the visible nature of skin lesions, PsO often has a detrimental impact on patients’ quality of life. This significant psychological burden necessitates comprehensive mental health support as part of disease management [18]. Socially, patients with PsO frequently face stigmatization and discrimination due to the conspicuous nature of their skin lesions. This social stigma often leads to isolation and diminished social interactions, adversely affecting QoL in its psychosocial dimension [19,20]. The level of independence is measured, focusing on an individual’s ability to perform daily activities and maintain a certain degree of autonomy [21]. Social relationships are evaluated by looking at personal connections, social support and involvement, and sometimes sexual activity. Environmental factors should also be considered to understand the impact of the surroundings on the individual’s life, including their safety, the quality of their home environment, their financial resources, whether they have access to healthcare, and their opportunities for recreation [22]. A QoL index should be a comprehensive measure designed to evaluate an individual’s overall well-being and life satisfaction in various dimensions, such as physical health, psychological state, level of independence, social relationships, and environmental factors. Examples of QoL indexes include the WHO-QoL (World Health Organization Quality of Life) and the SF-36 (Short-Form Health Survey). The WHO-QoL is a broad assessment that measures various components across different cultures and populations [23]. The SF-36, on the other hand, evaluates physical and mental health across eight domains, including physical functioning, bodily pain, general health perceptions, vitality, social functioning, and mental health [24]. PROs provide a holistic assessment, encompassing both visible symptoms and less overt impacts on QoL, and facilitate treatment evaluation by measuring improvements in symptoms, functional ability, and overall well-being [25]. This patient-centered approach supports tailored treatment plans and enhances shared decision-making between patients and healthcare providers [26]. In PsO, PROs capture the multidimensional effects on patients’ lives directly from their perspective. Among the most used PROs are DLQI and WHO-5. The DLQI consists of ten questions covering different aspects of daily life, such as symptoms and feelings, daily activities, leisure, work, personal relationships, and treatment. Each question is scored on a scale from 0 to 3, where 0 indicates no impairment and 3 indicates severe impairment. The total score ranges from 0 to 30, with higher scores indicating a greater impact on QoL [27]. Moreover, improvements in DLQI scores following treatment have been associated with better clinical outcomes and increased patient satisfaction [28]. WHO-5 has been recently introduced between PROs and consists of five positively worded items that assess aspects such as positive mood, vitality, and general interest over the past two weeks. Each item is scored on a Likert scale ranging from 0 (at no time) to 5 (all the time), with higher scores indicating better well-being. The total score ranges from 0 to 100 [29]. The results of our study corroborate findings from previous real-life studies, demonstrating a higher efficacy of 200 mg of tildrakizumab compared to 100 mg in terms of clinical efficacy and improvements in quality of life [16,17,23,24,25,30]. In our study, patient demographics were similar between the two groups, with minor variations in age and weight. Both groups experienced improvements in quality of life as assessed by the DLQI and the WHO-5. However, the group receiving 200 mg exhibited more pronounced and rapid reductions in both DLQI and WHO-5 scores. The WHO-5 showed a faster improvement in well-being at 4, 16, and 28 weeks compared to the DLQI, indicating its higher sensitivity to changes in mental well-being and overall quality of life. These findings align with other studies that have highlighted the responsiveness of WHO-5 in chronic disease management, including PsO. For instance, a study by Fredriksson and Pettersson emphasized the impact of effective PsO treatments on patients’ psychological well-being, corroborating the rapid improvements in WHO-5 scores observed in our study [31]. Similarly, Finlay and Khan demonstrated that QoL improvements in PsO patients are critical for overall treatment success, as reflected by reductions in DLQI scores in our study [27]. The safety profile of the treatment is crucial in managing chronic conditions like psoriasis, and in this study, no significant differences in side effects were reported between the two groups, underscoring the importance of maintaining patient well-being throughout therapy. Notably, there were no reported injection site reactions or major cardiovascular events, which are often concerns associated with long-term biologic treatments. In the G100 group, 2 out of 15 patients (13.3%) experienced mild side effects, specifically nausea and headaches, following the second dose, while in the G200 group, only 1 out of 15 patients (6.7%) reported diarrhea at week 4. Importantly, these side effects were manageable, and none of the patients discontinued therapy as a result, highlighting the overall tolerability of tildrakizumab. Ensuring that treatments are both effective and safe is essential, and the absence of severe adverse events in this study reinforces the potential of tildrakizumab as a viable long-term option for managing plaque psoriasis. This is consistent with phase III clinical trials that also reported minimal serious adverse events. The reSURFACE 1 and reSURFACE 2 trials highlighted the favorable safety profile of tildrakizumab, with low incidence rates of adverse events comparable to those in our study. When comparing the efficacy of tildrakizumab to other biologics, such as adalimumab and ustekinumab, data suggest that tildrakizumab, particularly at the 200 mg dosage, provides comparable if not superior improvements in both PASI scores and quality of life measures [32,33]. A head-to-head comparison study by Papp et al. found that tildrakizumab offered significant benefits over etanercept, particularly in terms of sustained QoL improvements as measured by DLQI [34]. The rapid improvement in WHO-5 scores reflects not only physical relief but also a substantial psychosocial benefit, enhancing patients’ overall QoL. Indeed, PsO significantly impacts psychological well-being due to its chronic and visible nature, leading to stigma and reduced social interactions; hence, the improvement in WHO-5 scores underscores the broader impact of effective PsO treatment on mental health. This is supported by research from Topp et al., which demonstrated that the WHO-5 is a reliable and valid measure of well-being in patients with chronic conditions, including PsO [12]. The WHO-5 and DLQI questionnaires serve different purposes in assessing patients’ quality of life and have distinct structures and focuses. The DLQI consists of 10 questions: 1 addressing subjective well-being, 1 focusing on symptoms, and the remaining 8 relating to daily activities. Each of these eight activity-related questions includes an optional “not relevant” response, which is scored as zero. This scoring method means that low DLQI scores may not accurately reflect the severity of the disease, as they can result from patients finding many activities irrelevant rather than experiencing minimal impact from the condition. In contrast, the WHO-5 consist of just five questions, all framed positively to capture the patient’s emotional state. None of these questions specifically addresses daily activities. This positive framing and focus on well-being provide a clear and direct measure of the patient’s emotional health. When evaluating scores, the DLQI exhibits a negative trend where lower scores indicate an improvement in QoL. On the other hand, the WHO-5 follows a positive trend, with higher scores representing better QoL. This positive trend makes the WHO-5 more intuitively aligned with improvements in patient well-being, as an increase in score directly correlates with enhanced QoL. Given these differences, the choice between the two partly depends on the context of the assessment. The DLQI is detailed and covers various aspects of daily life, making it useful for understanding the practical impacts of dermatological conditions. However, its scoring system and the potential for low scores not reflecting true disease severity can be limiting. The WHO-5, with its concise focus on emotional well-being and straightforward positive scoring, may be preferred for its simplicity and clear reflection of improvements in overall quality of life. For a holistic assessment that balances emotional well-being with functional impact, a combination of both questionnaires might be the most comprehensive approach. Indeed, combining the WHO-5 and DLQI questionnaires to create a complete assessment tool for quality of life involves integrating their strengths to provide a holistic view of the patient’s well-being [35]. We suggest administering both the WHO-5 and DLQI questionnaires sequentially during patient evaluations, beginning with the WHO-5 to gauge the patient’s overall emotional well-being, followed by the DLQI to assess the specific impacts on daily activities and symptoms.

## 5. Conclusions

In conclusion, this study reinforces the higher clinical efficacy and QoL improvements associated with tildrakizumab doses of 200 mg compared to 100 mg, with the WHO-5 proving to be a more sensitive and responsive tool than the DLQI with regard to capturing changes in well-being. In particular, the WHO-5 is more sensitive when determining the disease’s impact on patients’ quality of life compared to the DLQI. It is more manageable, with only five questions, and provides a more specific insight into patients’ feelings, making it a likely superior tool for assessing mental well-being. Our findings further support the use of tildrakizumab 200 mg as a potent therapeutic option for patients with moderate to severe PsO, providing significant benefits without an increased risk of adverse effects [36]. The study’s limitations include its monocentric design and small sample size, especially considering the recent introduction of double dosing in Italy. Future research should continue to explore the long-term outcomes of tildrakizumab treatment and its comparative effectiveness against other biologic therapies in diverse patient populations. Additionally, further studies should consider incorporating more detailed PROs to better understand the full impact of treatment on QoL.

## Data Availability

The data that support the findings of this study are available from the corresponding author upon reasonable request.
